# Interferon Regulatory Factor 8 Regulates Pathways for Antigen Presentation in Myeloid Cells and during Tuberculosis

**DOI:** 10.1371/journal.pgen.1002097

**Published:** 2011-06-23

**Authors:** Jean-François Marquis, Oxana Kapoustina, David Langlais, Rebecca Ruddy, Catherine Rosa Dufour, Bae-Hoon Kim, John D. MacMicking, Vincent Giguère, Philippe Gros

**Affiliations:** 1 Department of Biochemistry, McGill University, Montréal, Canada; 2 Laboratoire de Génétique Moléculaire, Institut de Recherches Cliniques de Montréal, Montréal, Canada; 3 Goodman Cancer Center, McGill University, Montréal, Canada; 4 Section of Microbial Pathogenesis, Boyer Center for Molecular Medicine, Yale University School of Medicine, New Haven, Connecticut, United States of America; The Jackson Laboratory, United States of America

## Abstract

IRF8 (Interferon Regulatory Factor 8) plays an important role in defenses against intracellular pathogens, including several aspects of myeloid cells function. It is required for ontogeny and maturation of macrophages and dendritic cells, for activation of anti-microbial defenses, and for production of the Th1-polarizing cytokine interleukin-12 (IL-12) in response to interferon gamma (IFNγ) and protection against infection with *Mycobacterium tuberculosis*. The transcriptional programs and cellular pathways that are regulated by IRF8 in response to IFNγ and that are important for defenses against *M. tuberculosis* are poorly understood. These were investigated by transcript profiling and chromatin immunoprecipitation on microarrays (ChIP-chip). Studies in primary macrophages identified 368 genes that are regulated by IRF8 in response to IFNγ/CpG and that behave as stably segregating expression signatures (eQTLs) in F2 mice fixed for a wild-type or mutant allele at *IRF8*. A total of 319 IRF8 binding sites were identified on promoters genome-wide (ChIP-chip) in macrophages treated with IFNγ/CpG, defining a functional G/AGAAnTGAAA motif. An analysis of the genes bearing a functional IRF8 binding site, and showing regulation by IFNγ/CpG in macrophages and/or in *M. tuberculosis*-infected lungs, revealed a striking enrichment for the pathways of antigen processing and presentation, including multiple structural and enzymatic components of the Class I and Class II MHC (major histocompatibility complex) antigen presentation machinery. Also significantly enriched as IRF8 targets are the group of endomembrane- and phagosome-associated small GTPases of the IRG (immunity-related GTPases) and GBP (guanylate binding proteins) families. These results identify IRF8 as a key regulator of early response pathways in myeloid cells, including phagosome maturation, antigen processing, and antigen presentation by myeloid cells.

## Author Summary

IRF8 is a member of the Interferon Regulatory Factor family that is expressed in myeloid cells such as macrophages and dendritic cells and that activates or represses gene transcription upon stimulation with interferon gamma (IFNγ), lipopolysaccharide (LPS), and other microbial stimuli. IRF8 plays an important role in several aspects of myeloid cells, including differentiation and maturation of early progenitor cells, expression of intrinsic anti-microbial defenses, and production of the interleukin-12 (IL12) cytokine, which is essential for priming of early T cell– mediated immune response. IRF8 mutant mice are susceptible to a number of intracellular infections including pulmonary tuberculosis. The transcriptional and cellular pathways regulated by IRF8 and essential for resistance to infections were studied by a combination of genome-wide methods, including transcriptional profiling and chromatin immunoprecipitation (ChIP-chip). These studies identified phagosome maturation, antigen processing, and antigen presentation as critical pathways in early host–pathogen interactions regulated by IRF8 in macrophages exposed to IFNγ/CpG and in lung tissues infected with *Mycobacterium tuberculosis*.

## Introduction

The defense mechanisms of mononuclear phagocytes that are circumvented by successful intracellular pathogens are poorly understood [1]. Genes and proteins in these pathways may represent valuable targets for therapeutic interventions in the corresponding diseases. Such host defense mechanisms can manifest themselves as genetic determinants of innate resistance or susceptibility to infections in human populations [2], [3], and in corresponding animal models of experimental infections [4], [5]. Forward genetic studies of naturally occurring or experimentally induced mutations in mice may identify such genes and proteins [5], [6], which relevance to the corresponding human infection can be established in parallel studies of human populations from areas of endemic disease [2], [5], [6].

In inbred mouse strains, susceptibility to infection with several intracellular pathogens including *Mycobacterium*, *Salmonella* and *Leishmania*, is determined in part by the natural resistance-associated macrophage protein 1 (*Nramp1*) gene (*Slc11a1*). In resistant mice, Slc11a1 functions as an efflux pump for Fe^2+^ and Mn^2+^ ions at the membrane of microbe-containing phagosomes formed in macrophages, thereby restricting microbial access to these essential nutrients [7]. In humans, polymorphic variants at or near *SLC11A1* have been associated with differential susceptibility to mycobacterial infections including tuberculosis, leprosy, and Buruli ulcer [6]. In addition, monocytes derived from individuals bearing *SLC11A1* alleles associated with tuberculosis susceptibility in field studies, display reduced functional activity of the SLC11A1 protein [8]. A search for genetic modifiers of the protective effect of *Slc11a1* identified the BXH2 mouse strain as highly susceptible to *Mycobacterium bovis* (BCG; bacillus Calmette-Guérin) infection despite presence of resistance-associated *Slc11a1* alleles (*Slc11a1^Gly169^*) [9]. By positional cloning, we determined that susceptibility to infection in BXH2 is caused by a mutation (*R294C*) in the interferon regulatory factor 8 (IRF8) (Ensembl:ENSMUSG00000041515) [9].

IRF8 is one of 9 members of the Interferon Regulatory Factor (IRF) family. IRF8 has a DNA binding domain (DBD; 120 a.a) of the helix-turn-helix type that binds to ISRE (Interferon Stimulated Response Elements) sites present in the proximal promoters of type II IFN-regulated genes. IRF8 also has an IRF association domain (IAD) that serves as a recruitment module for other transcription factors. IRF8 is expressed primarily in macrophages and dendritic cells, but is also detected in T and B lymphocytes [10]; and upon stimulation with interferon gamma (IFNγ), lipopolysaccharide (LPS), pathogen-associated molecular patterns (PAMPs) and other microbial stimuli, IRF8 binds to ISREs in association with other members of the IRF (e.g. IRF1), or ETS (e.g. PU.1, TEL) families, or other hetero-dimerization partners, to activate or repress gene expression in these cells [11]. The IRF8-PU.1 heterodimer leads to the activation of genes containing ETS-IRF composite element (EICE, GGAAnnGAAA), the ETS-IRF response element (EIRE, GGAAAnnGAAA) or to the IRF-ETS composite sequence (IECS, GAAAnn(n)GGAA) [12]. IRF8 plays an important role in several physiological aspects of myeloid cells development and function. IRF8 drives differentiation of myeloid progenitors towards mononuclear phagocytes, while positively regulating apoptosis of the granulocytic lineage [11], [13]. Macrophages from IRF8-deficient mice remain immature, including altered expression of intrinsic macrophage anti-microbial defenses [11], and are susceptible to *ex vivo* infection with *M. bovis* [14], *Salmonella typhimurium* [14], and *Legionella pneumophila* [15]. IRF8-deficient mice also show a profound defect in dendritic cells (DCs), as they lack both CD11c^+^CD8α^+^ DCs and pDCs [16]. In addition, the small number of CD11c^+^CD8α^+^ and CD8α^−^ DCs present in these mice remain immature and fail to up-regulate co-stimulatory molecules and to produce key cytokines in response to microbial products [16]–[18]. In addition, IRF8 is required for Th1 polarization of early immune response [11]. This cooperation between antigen presenting cells (APCs) and T/NK cells, involves IFNγ binding to its receptor (IFNγR) which causes STAT1 (signal transducers and activators of transcription) activation. STAT1 trans-activates IRF8 expression leading to IL-12p40 production by dendritic cells, and engagement of the interleukin 12 receptor (IL12R) on Th1 cells further amplifies IFNγ production [11]. IRF8 binds to the promoter regions, and is required for activation of IL-12p40 [19], [20], IL-12p35 and IL-18 genes in DCs in response to IFNγ [11], [19], [20]. *IRF8^−/−^* mice do not produce IL-12p40, lack Th1 polarization (absence of antigen specific CD4^+^, IFNγ producing T cells), and are susceptible to *in vivo* infection with intracellular pathogens [19], [21]–[24].

We have shown that the *IRF8^R294C^* isoform of BXH2 behaves as a partial loss-of-function which is associated with impaired IL-12p40 production by BXH2 splenocytes, and loss of trans-activation of a IL-12p40 reporter construct *in vitro*. The *IRF8^R294C^* mutation results in increased *M. bovis* (BCG) multiplication both early and late during infection, with uncontrolled replication linked to inability to form granulomas in infected liver and spleen. The *IRF8^R294C^* mutation also causes susceptibility to *S. typhimurium* to a level comparable to that seen for mice lacking functional *Nramp1* or *Tlr4* (Toll-like receptor 4), and impairs innate and adaptive immune defenses against the blood-stage malarial parasite *Plasmodium chabaudi* AS [25]. BXH2 mice are also extremely susceptible to aerosol infection with *Mycobacterium tuberculosis*, showing uncontrolled intracellular pathogen replication in lung macrophages, impaired granuloma formation, rapid dissemination of the infection to distant sites, and rapid necrosis of infected tissues, and early death. There was complete absence of IL-12p40 induction, severely reduced IFNγ production, and impaired T cell priming in the lungs of infected BXH2, highlighting the critical role of IRF8 in this response [26]. These studies have identified IRF8 as a key regulator of host defenses against Mycobacteria.

In this study, we have used transcript profiling with microarrays and chromatin immunoprecipitation (ChIP) hybridization on genomic DNA arrays (ChIP-chip) in macrophages from normal and IRF8-deficient mice, to systematically identify genes transcriptionally regulated by IRF8 a) during ontogeny and maturation of macrophages, and b) in response of these cells to combined exposure to IFNγ and Tlr9 (Toll-like receptor 9) ligand (CpG), and c) during pulmonary tuberculosis *in vivo*. In these studies, we incorporated an experimental strategy based on the co-segregation of IRF8-dependent differential gene expression in macrophages from [BALB/c×BXH2] F2 animals selected for homozygosity for either wild-type (wt; *IRF8^R294^*) or mutant (*IRF8^C294^*) *IRF8* alleles. These studies have identified a critical role for IRF8 in regulating expression of genes and associated cellular pathways responsible for early interaction with pathogens, phagosome maturation, antigen processing and antigen presentation to CD4^+^ and CD8^+^ T cells.

## Results

### Identification of IRF8-Dependent eQTLs Segregating in F2 Animals

To identify transcriptional targets of IRF8 that play a role in a) macrophage maturation, and b) in activation in response to IFNγ and microbial products, we used transcript profiling to compare RNA expression in macrophages bearing either a wild-type (wt) or a mutant allele at *IRF8* (R294C). For this, we used bone marrow-derived macrophages (BMDMs) from individual [BALB/c×BXH2] F2 mice of mixed genetic background but that were identified as homozygote for either wt (*IRF8^R294^*) or mutant (*IRF8^C294^*) *IRF8* alleles. This strategy [27] is based on the observation that complex gene expression profiles (eQTLs) caused by a null mutation at a specific gene show extremely robust segregation in F2 animals [28], congenic strains [29] or recombinant congenic lines [30] derived from parental strains bearing wt and mutant alleles at the gene of interest. Gene expression profiles detected in common in macrophages from F2 animals of either wt or mutant *IRF8* genotypes but that show mixed genetic background (C57BL/6J, C3H/HeJ, BALB/cJ), can distinguish true IRF8-dependent effects from irrelevant ones caused by differences in genetic background of the two parental mouse strains. This strategy is well suited to study eQTLs caused by absence versus presence of a transcription factor such as IRF8. In this approach, individual F2 mice (6 samples per experimental group) are used both as biological and technical replicates, to increase the stringency of the analysis. BMDMs from wt and *IRF8^C294^* F2 mice were stimulated or not with IFNγ/CpG, and RNA was isolated and used for transcript profiling.

### IRF8-Dependent Transcript Profiles Associated with Macrophage Maturation

**Figure 1:**
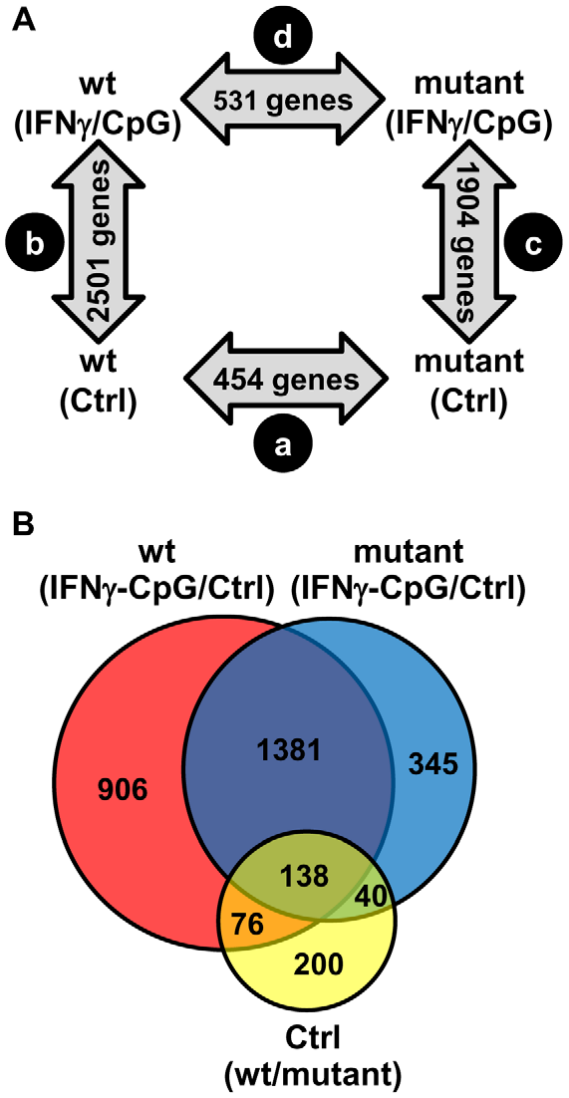
Pairwise analysis of transcriptional responses of wt and IRF8 mutant BMDMs at basal level and following exposure to IFNγ/CpG. BMDMs RNA was obtained from individual wt and IRF8 mutant F2 mice either prior to (unstimulated control) or 3 hrs following stimulation with IFNγ/CpG (6 samples per experimental group; 24 samples in total), and hybridized to microarrays. The 3 hrs IFNγ/CpG-stimulated macrophage cultures were initially primed with IFNγ (50 U/ml) for 18 hrs. (A) A closed-loop strategy was applied to monitor differences in transcript abundance between the four experimental groups; (comparison a) mutant control versus wt control; (comparison b) wt stimulated versus wt control; (comparison c) mutant stimulated versus mutant control; (comparison d) mutant stimulated versus wt stimulated. The numbers of significantly modulated transcripts identified for each single pairwise comparison are indicated within the gray arrows. (B) A Venn diagram analysis of the pairwise comparisons a, b, and c revealed considerable overlaps in the lists of transcripts which level of expression is affected by the IRF8 alleles and the IFNγ/CpG stimulation.

IRF8 plays a critical role in maturation of monocytes, macrophages and dendritic cells, and mice bearing mutations at *IRF8* have defects in these cell types [11]. To identify IRF8 transcriptional targets that may play a role in maturation of the myeloid lineage, we compared transcript profiles in resting BMDMs from wt and *IRF8* mutant F2 mice (Figure 1A). A pairwise analysis (*t* test p value<0.05; fold change ≥1.5X) identified a total of 454 genes differentially expressed in an IRF8-dependent fashion in these cells at basal level (Table S1). Of these 454 genes, 219 were more highly expressed in wt cells, while 235 were more highly expressed in mutant BMDMs (Table S1). Hierarchical clustering of the 454 genes according to expression pattern similarities in the 12 independent microarrays readily separated the 6 individual wt mice from the 6 individual mutant mice (data not shown), illustrating the robustness of the approach. A gene ontology (GO) report on these 219 and 235 genes separately, revealed that 39 (17.8%) and 45 (19.1%) of them were associated with ‘response to stimulus’, representing the most abundant group (data not shown). Additional enriched gene clusters included GO-terms such as immune system development, immune system process, response to stress, intracellular signaling cascade, immune response, defense response, transcription, and others. Genes most positively regulated by IRF8 in resting cells included genes involved in a) antigen processing and presentation (*CD74*, *H2-AbI*, *H2-Eb1*, *H2-Ea*), b) cytokines and chemokines production and signaling (*Cxcl14*, *Cxcl16*, *Socs2*, *Ciapin1*, the *C1q* complex, *Il17ra*), c) growth regulation (*Csf3r*), d) tissue remodeling (*Timp1*, *Vcam1*), and e) rapid response to microbial insults (*Mx1*, *Ifitm1*, *Tnfaip3*, *Ly86*) [see Table S1 for annotation]. Together these genes may correspond to direct IRF8 targets or may represent markers of maturation differentially expressed in response to the block caused by loss of IRF8 function in BMDMs.

### IRF8-Dependent Transcript Profiles Associated with Macrophage Activation by IFNγ/CpG

To systematically identify IRF8 targets that are important for IFNγ-induced macrophage activation, we compared gene expression profiles obtained in BMDMs from wt and *IRF8* mutant F2 mice following exposure to IFNγ/CpG (stimulated versus control). A first pairwise analysis (*t* test p value<0.05; fold change ≥1.5X) identified a total of 2501 (1247 induced; 1254 repressed) and 1904 (828 induced; 1076 repressed) genes significantly regulated by IFNγ/CpG in wt and *IRF8* mutant mice, respectively (Figure 1A). A subset of these genes (76 genes in wt only, 40 genes in *IRF8* mutant only, and 138 genes in both groups) were also regulated by IRF8 at the basal level, in the absence of IFNγ/CpG stimulation (Figure 1B and Table S2).

**Figure 2:**
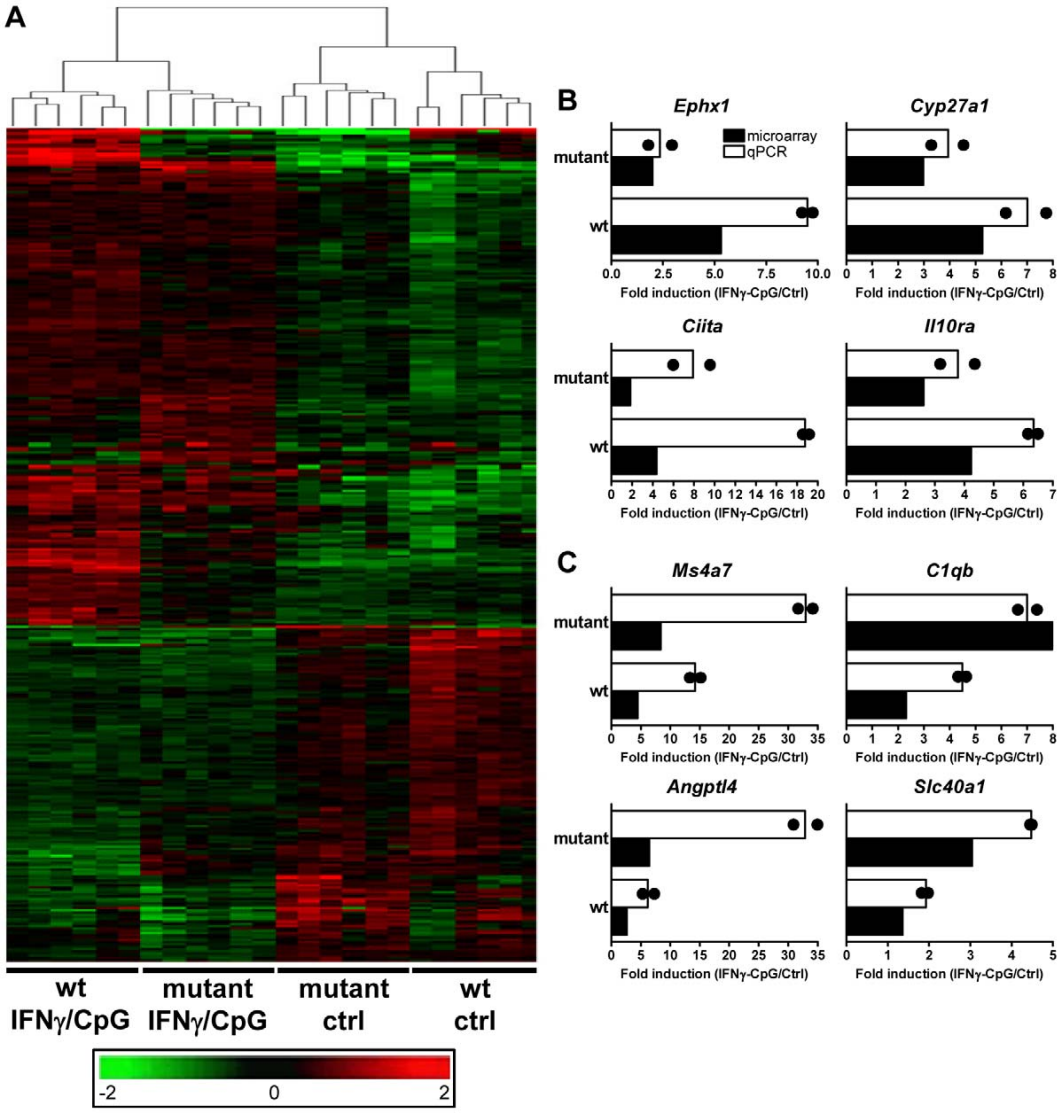
Transcriptional programs elicited by IFNγ/CpG exposure in wt and IRF8 mutant F2 mice. BMDMs RNA was obtained from individual wt and IRF8 mutant F2 mice either prior to (unstimulated control) or 3 hrs following stimulation with IFNγ/CpG (6 samples per experimental group; 24 samples in total), and hybridized to microarrays. (A) By using a 2×2 interaction Anova analysis, 368 genes were recognized to be significantly differentially modulated by IRF8 in response to IFNγ/CpG exposure between the wt and IRF8 mutant cells. The expression profiles are ordered by hierarchical clustering; the genes, illustrated by their specific signal intensities (Log_2_ scale) are displayed as rows and individual mouse samples/conditions as columns. Red coloring signifies high level of expression; green coloring denotes low level of expression. The dendrogram illustrates the clustering of the samples according to expression pattern similarities. RNA samples (6 per experimental group) used for this transcriptional profiling analysis were pooled and used for qPCR validation. *Ephx1*, *Cyp27a1*, *Ciita*, and *Il10ra* were selected as genes positively affected by the presence of functional IRF8 following IFNγ/CpG exposure (B), while *Ms4a7*, *C1qb*, *Angptl4*, and *Slc40a1* were selected as genes negatively affected by the presence of a functional IRF8 following IFNγ/CpG exposure (C). The ratios of expression (IFNγ/CpG-stimulated versus unstimulated control), represented by fold induction, were calculated for the wt and IRF8 mutant mice separately (white bars), and compared to the corresponding microarray results (black bars). The black dots indicate the qPCR values obtained for each replicate. The microarray results were statistically significant according to Anova analysis (*t* test p value of 0.05 and a fold-change cutoff of 1.5X). *Hprt* was used to standardize the mRNA levels of target genes for qPCR.

Secondly, and to take into account possible IRF8-dependent expression differences at basal level, we carried out a two-way (2×2 interaction) Anova analysis [29]. In this analysis, expression levels before and after IFNγ/CpG treatment are calculated and expressed as ratios, and a statistical analysis is conducted to identify genes which ratio of expression are affected by IRF8 [wt, stimulated versus unstimulated; compared to *IRF8* mutant, stimulated versus unstimulated] with a *t*-test p value<0.05 and a fold change ≥1.5X. This comparison identified 368 genes that were significantly regulated by IRF8 in response to IFNγ/CpG (Figure 2A and Table S3). Hierarchical clustering according to expression pattern similarities not only distinguished the control from treated groups (IFNγ/CpG), but also separated wt from *IRF8* mutant BMDMs and this for both conditions (dendrogram in Figure 2A). A subset of 80 genes showed particularly robust IRF8 dependence in expression in response to IFNγ/CpG, while showing no significant IRF8-dependent effects at basal levels (indicated in bold in Table S3). This list contained many genes known to play a key role in several aspects of macrophage function, including a) cytokine-cytokine receptor interaction (*Ccl8*, *Ccr3*, *Il13ra1*), b) antigen presentation (*H2-DMb2*, *Ciita*), c) tissue remodeling (*Angptl4*, *Col18a1*, *Mmp13*), d) detoxification (*Cyp27a1*, *Cyp4f18*, *Cyp51*, *Por*, *Ephx1*), e) cell surface receptors (*Igh-6*, *Tfrc*) and adhesion molecules (*Siglec1*), and *Irf4*, a member of the IRF family know to functionally interact with IRF8 to regulate gene expression [11]. A subset of 8 transcripts (*Ephx1*, *Cyp27a1*, *Ciita*, *Il10ra*, *Ms4a7*, *C1qb*, *Angptl4*, and *Slc40a1*) strongly induced by IFNγ/CpG in an IRF8-dependent manner, were selected for further validation by quantitative PCR (qPCR). For all the genes tested, we observed an excellent correlation between the level and degree of differential expression initially detected by transcript profiling and results from qPCR analysis (Figure 2B and 2C).

### Identification and Characterization of IRF8 Binding Sites in Activated Macrophages by Chromatin Immunoprecipitation (ChIP-Chip)

**Figure 3:**
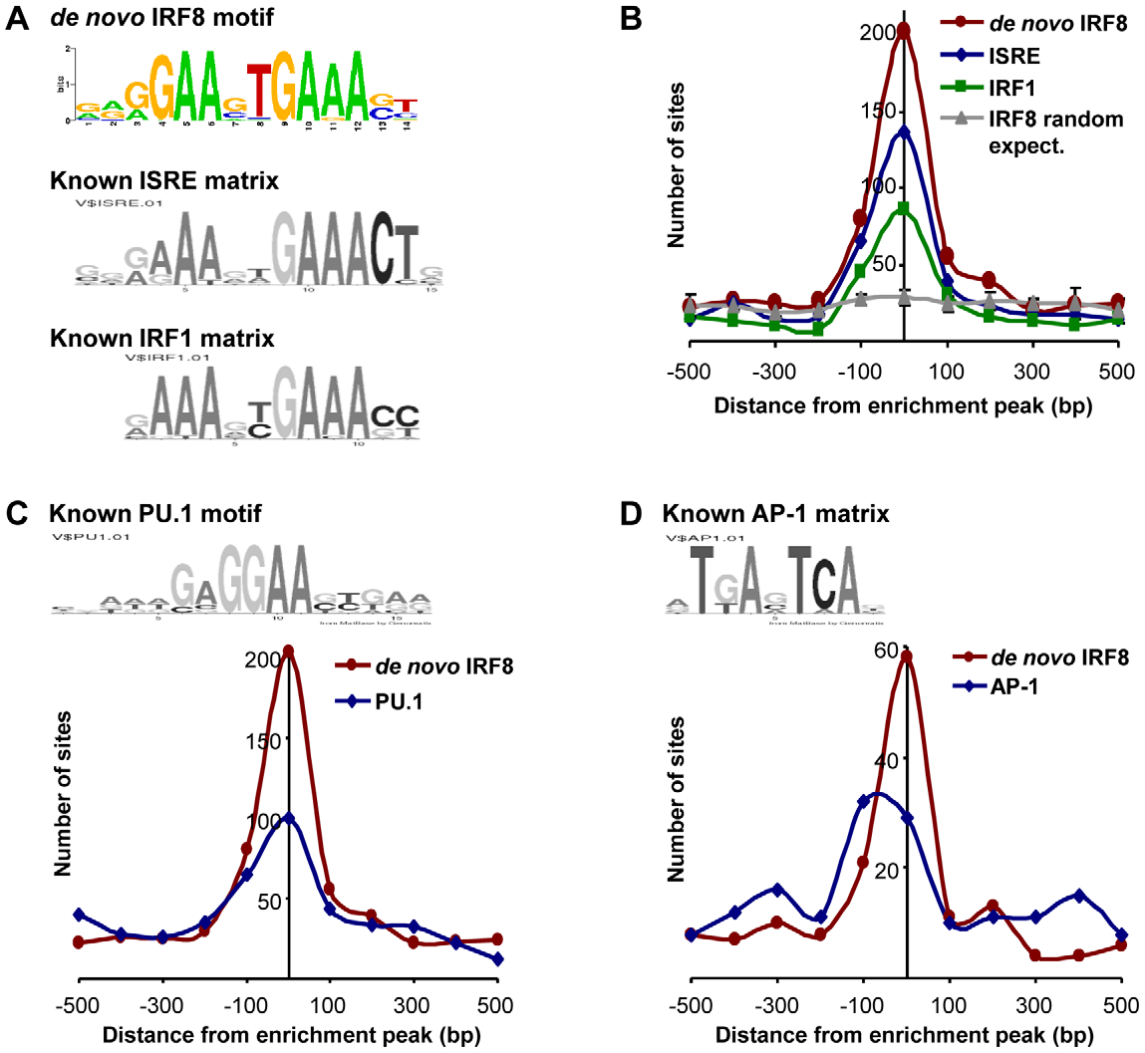
Transcription factor binding motif analyses on IRF8 ChIP-chip binding sites. IRF8 chromatin immunoprecipitated from IFNγ activated macrophages was hybridized to Agilent promoter tiling array (ChIP-chip). After normalization and statistical analysis, we identified 319 IRF8 binding sites with a threshold of 2 fold enrichment and *p* value≤0.001 relative to a non-specific control antibody ChIP. (A) *De novo* binding motif analysis was carried out on 500 bp sequence flanking the IRF8 sites. The MEME algorithm returned an IRF-like motif as the top motif with a high score. The known ISRE and IRF1 motifs are from the Genomatix MatBase database. (B) The 319 IRF8 binding sites were queried for the IRF8 *de novo* binding motif and for the ISRE and IRF1 motif shown in A. The position of the *in silico* found sites was plotted relative to the ChIP-chip IRF8 binding peak. They all cluster over the peak center, whereas there is no IRF8 motif enrichment in a set of randomly chosen sequences. (C) The PU.1 (ETS family member) motif shows a clear colocalization with the IRF8 *de* novo motif, as reported by the *in silico* analysis. (D) The AP-1 motif is also enriched.

Transcript profiling analyses revealed that IRF8 intervenes in a complex transcriptional network. To identify which genes in this network are direct IRF8 transcriptional targets of (as opposed to secondary targets), we hybridized IRF8-bound chromatin obtained by immunoprecipitation (ChIP) from cultured macrophages treated with IFNγ/CpG to Agilent promoter tiling arrays (ChIP-chip). Following normalization and statistical analysis, we identified 319 IRF8 binding events corresponding to 333 different genes (Table S4). These binding sites were selected for a) a minimum of 2-fold enrichment over control ChIP carried out using non-immune serum, and b) a p value≤0.001. In this list, we validated IRF8 recruitment to *Ifnβ* promoter by ChIP-qPCR (data not shown). Moreover, this list contains several published IRF8 binding sites (*Tlr4*, *Oas2*, *Cybb*, *Ifitm3*, *Etv3*, *Lyz* and *Tlr9*) and shows a significant 43% overlap with the recently published IRF8 ChIP-chip study performed on chromatin from human monocytes [31]. To analyse closely the IRF8 binding sequence, we determined the chromosomal position for the center of each binding peak, and extracted 500 bp of peak flanking sequence using the mouse mm8 genome assembly. These sequences were queried for *de novo* motif discovery with different algorithms (MEME, MDscan and AlignAce), and all produced the same IRF8 DNA binding motif (G/AGAAnTGAAA) as the top matrix (Figure 3A and Figure S1A) [32]–[34]. This highly significant motif (MEME E-value = 8.3^−385^) is in agreement with the known Transfac database IRF8 binding motif (ICSBP_M00699; Figure S1B), although there is no requirement for the 3′ CTG bases that are more characteristic of the ISGF3 (Stat1/Stat2/IRF9) ISRE binding site (Figure 3A). This *de novo* binding site is closer to a standard IRF site which is characterized by a 2 nucleotide spaced tandem repeat of GAAA, with an important difference, the first base is mostly occupied by a guanine. A comparable GGAAnnGAAA motif was previously described as ETS-IRF composite element (EICE) [12]. However, the motif identified in the present study gives importance to a T placed in the sixth position. This *de novo* derived motif was found at least once within a 1000 bp segment of 87% (277 out of 319) of the IRF8 binding sites identified by ChIP-chip. A comparison of the *de novo* defined IRF8 site with known ISRE and IRF1 binding motif show they all cluster at the peak of enrichment, with the highest number for the *de novo* IRF8 site (Figure 3B).

Other predominant motifs were identified in our dataset by *de novo* analysis. Their similarity to known Transfac v11.3 database was assessed using the STAMP web-tool [35]. With the MatInspector motif search tool, we measured the fold enrichment of each *de novo* and known motifs occurrence in our dataset compared to similar sets of random sequences (Table S5). As expected, all the matrices from IRF family were enriched; ETS family motifs were also enriched because the GGAA sequence which forms part of their binding site (GAGGAA) is imbedded within the IRF8 binding site. We detected a strong association (∼50% of sites) between the *de novo* generated IRF8 motif and binding sites for PU.1, the major ETS factor in macrophages, with co-localization of the two sites at the binding peak (Figure 3C). In addition, we noted an enrichment of AP-1 sites: of the 277 IRF8 motif containing peaks, 73 (26%) also contain an AP-1 predicted site with a tendency of these sites to be centered at the peak of enrichment, although not as clearly as for PU.1 (Figure 3D).

**Figure 4:**
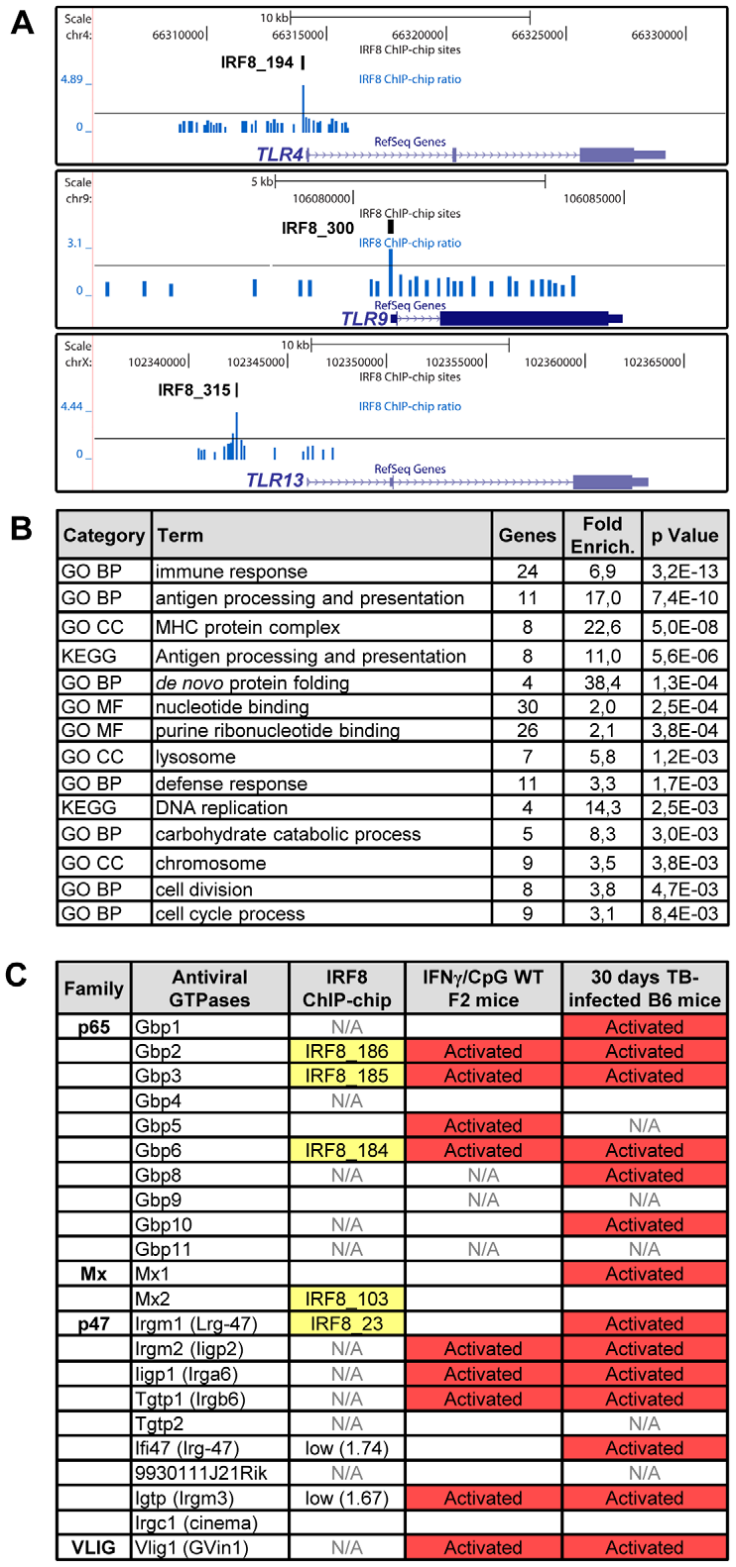
IRF8 regulates important functions of APC cells. (A) IRF8 ChIP-chip binding profiles extracted from the UCSC genome browser for Toll-like receptors (*Tlr4,9,13*); Black rectangles represent the significant binding peak and the blue bars correspond to ChIP-chip binding ratios. (B) Gene ontology (GO) and KEGG pathways analysis of the genes regulated by IFNγ-CpG treatment in F2 wt mice and having an IRF8 binding site in their proximity. The IRF8 targets genes are implicated in immune response, nucleotide binding and antigen processing and presentation. (C) Antiviral GTPases gene regulation summary. Association of known antiviral GTPases with IRF8 binding sites, IFNγ/CpG regulation in wt F2 mice on Illumina Mouse WG-6 v2.0 Expression BeadChip arrays and regulation after *M. tuberculosis* infection in B6 mice on Affymetrix oligonucleotides chips (Mouse Genome 430 2.0 array). N/A indicates that this was not assessed by the microarrays.

A Gene Ontology (GO) analysis with the DAVID (database for annotation, visualization and integrated discovery) web-tool for genes exhibiting an IRF8 binding peak detected by ChIP-chip revealed a strong enrichment for the “immune response” category (29 genes, p value = 5.3e10^−9^) (Table S6) [36], [37]. This list includes several genes encoding proteins involved in recognition, processing and presentation of antigens by antigen-presenting cells (APCs). Indeed, it includes members of the Toll-like receptors (TLR) family that play a crucial role in recognition of pathogen-associated molecular signatures, including *Tlr4* (interaction with LPS from Gram-negative bacteria), *Tlr9* (unmethylated CpG containing DNA) and *Tlr13* (vesicular stomatitis virus) (Figure 4A) [38]. KEGG (Kyoto encyclopedia of genes and genomes) pathway enrichment analysis identifies several genes that play a key role in antigen processing and presentation in dendritic cells and macrophages, including Class I and Class II MHC (major histocompatibility complex) molecules, as well as proteases, membrane transporters and structural proteins involved in generation, transport and loading of antigenic peptides onto Class I or Class II molecules (Figure 4B and Table S6). Finally, we also note an enrichment of IRF8 binding peaks in the GO term nucleotide binding. Strikingly, many of the genes contained in that list include members of the IFN-inducible GTPase superfamily, including the Gbp (guanylate binding proteins), Mx, and p47 (Irg; immunity-related GTPases) families which are involved in early innate immune response to intracellular infection in many cell types (Figure 4B, 4C and Table S6) [39]–[41].

### Identification of Direct IRF8 Targets That Are Regulated by IFNγ in Macrophages *In Vitro* and in the Lung during Pulmonary Tuberculosis

To identify direct functional targets of IRF8 in macrophages, we overlapped the list of IRF8 binding peaks (ChIP-chip) with the list of genes differentially regulated by exposure to IFNγ/CpG in macrophages from F2 mice bearing wt alleles at *IRF8*. This intersection included 145 direct IRF8 targets controlled by 111 IRF8 binding sites (Table S7). We also examined the overlap between IRF8 binding sites detected by ChIP-chip and the genes which expression in macrophages in regulated by IRF8 in response to IFNγ/CpG (from 2×2 Anova analysis, Figure 2A and Table S3). We found 21 genes that are regulated in this fashion and that harbour an IRF8 binding site in their vicinity. These genes represent transcriptional targets of IRF8 which expression is regulated by IFNγ/CpG in macrophages in an IRF8-dependent fashion. The vast majority of these 21 genes were included in the intersection detected between IRF8 binding sites and genes regulated by IFNγ/CpG in wt F2 macrophages (Table S7).

**Figure 5:**
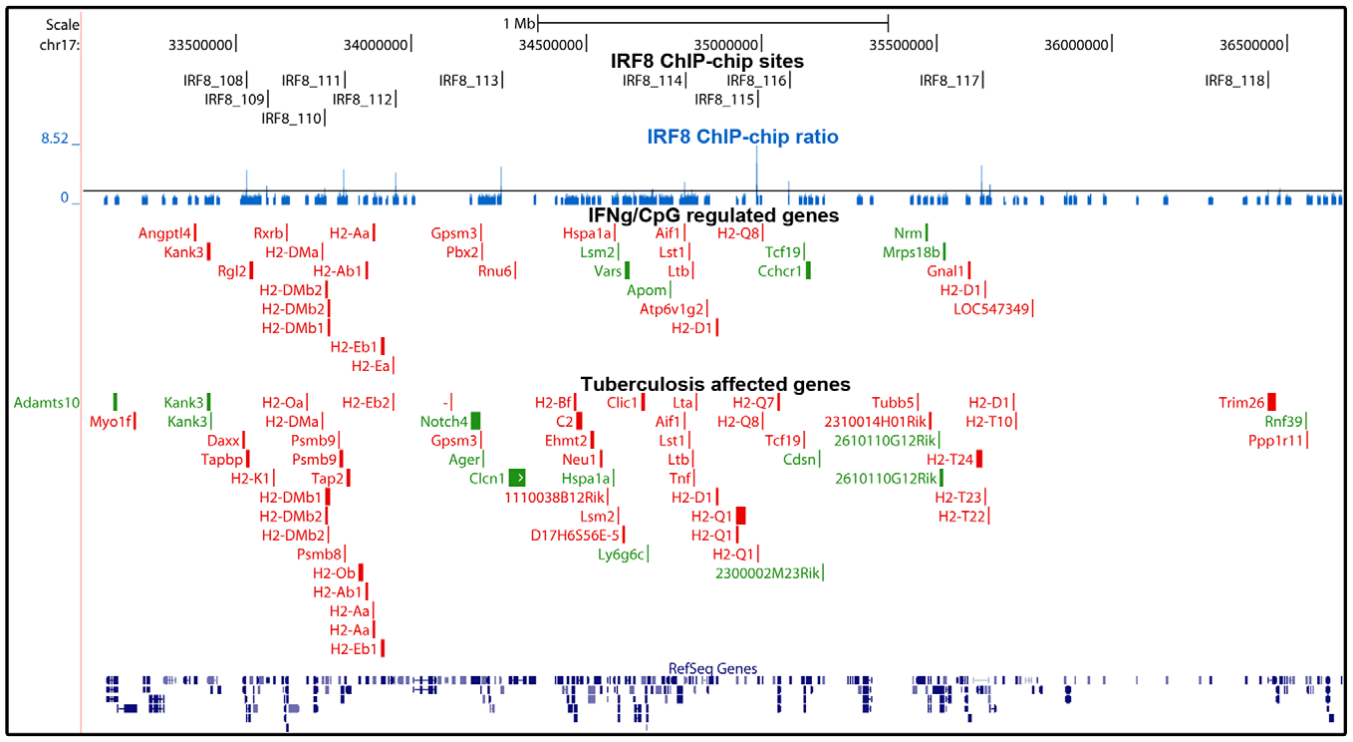
Enrichment of IRF8 targets within the boundaries of the MHC locus. IRF8 ChIP-chip binding profiles extracted from the UCSC genome browser for the MHC locus. The black rectangles represent the significant binding peak and the blue bars correspond to ChIP-chip binding ratios. Genes regulated by IFNγ/CpG and/or *M. tuberculosis* infection are represented by red (activation) and green (repression) boxes.

**Figure 6:**
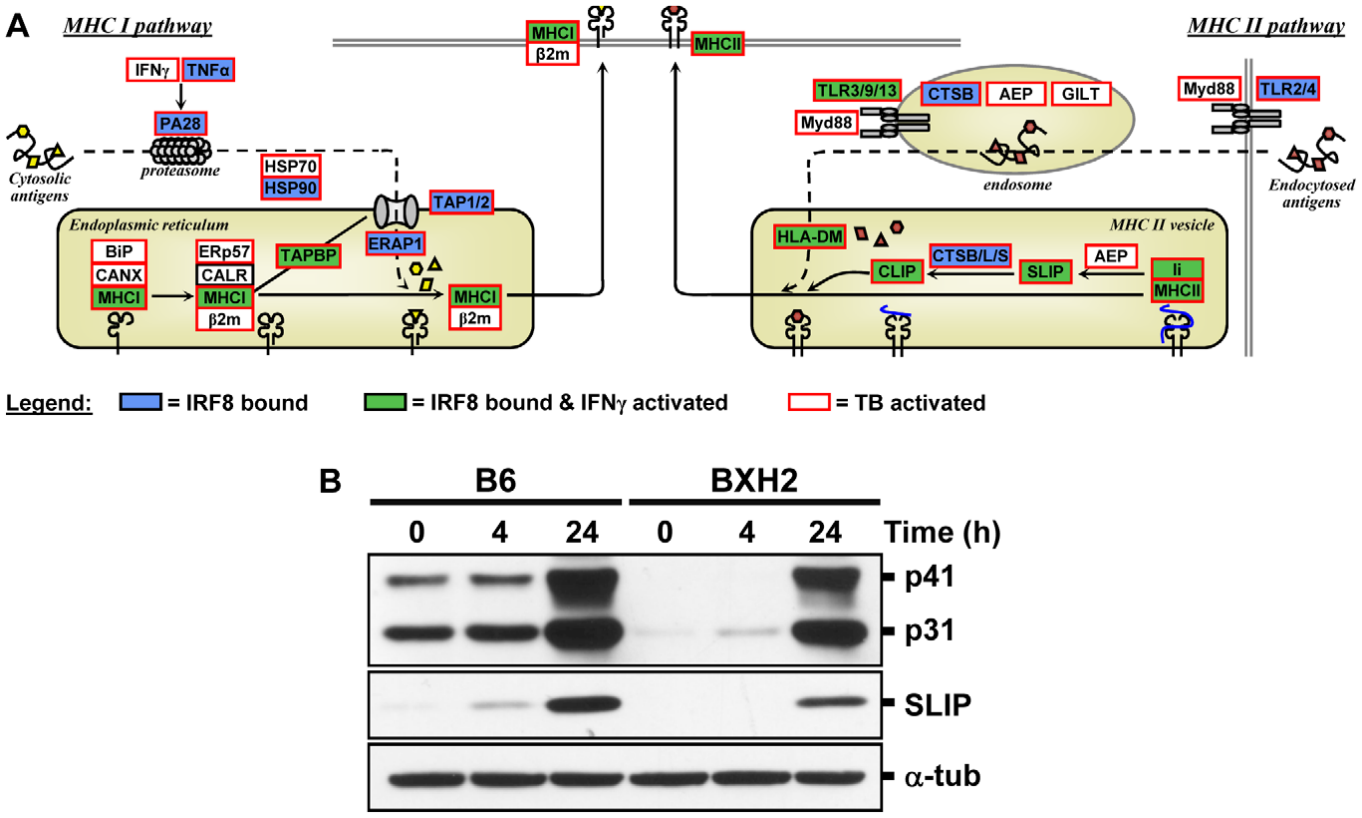
The IRF8 binding sites are strongly associated with antigen presenting cells function. (A) Schematic representation of the “antigen processing and presentation” pathway. Adapted from the KEGG pathway database [61], [62] and current literature [63]. Genes implicated in Class I and Class II MHC antigen processing and presentation were annotated for IRF8 binding (blue box), for activated by IFNγ-CpG (green box), and for activation by infection with *M. tuberculosis* (red outline). These results suggest that IRF8 is a key regulator of all steps of the antigen processing and presentation pathway. (B) Validation of Cd74 (Ii) at protein level. BMDMs total proteins were obtained from wt (B6) and IRF8 mutant (BXH2) mice either prior to (unstimulated control) or following stimulation with IFNγ/CpG (4 hrs and 24 hrs post-stimulation), separated in 10% SDS-PAGE and probed with In-1 mAb. The migration of intact p41 and p31 Ii, as well as SLIP Ii fragment (∼12-kD) is indicated. α-tubulin was used as a constitutively expressed internal control to normalize the protein levels.

We also investigated the relevance of IRF8 targets discovered by ChIP-chip, to host defenses against infections *in vivo*. IRF8 and IFNγ are required for protection against pulmonary infection with *M. tuberculosis* [26], and mice bearing mutations in either gene are hyper-susceptible to pulmonary tuberculosis [26], [42]. IRF8 is required for development of the dendritic cell lineages, IL-12 production by these cells (Th1 polarization of immune response), recruitment of T cells to the site of infection, macrophage activation and containment of infection by activated macrophages in granulomas [43]. To identify IRF8 targets that may play an important role in host defenses against pulmonary tuberculosis, we investigated which of the 319 IRF8 binding sites and associated genes are significantly regulated in the lungs of C57BL6/J (B6) mice 30 days following aerosol infection with *M. tuberculosis* (pairwise analysis of day 30 versus day 0 transcript profiles) [29]. An intersection of 213 IRF8 binding sites corresponding to 359 associated transcription units was detected in this analysis. Therefore, ∼2/3 of the identified IRF8 targets were found to be modulated during *M. tuberculosis* infection *in vivo*. In addition, there was considerable overlap between the list of IRF8 targets which expression was regulated by a) IFNγ/CpG stimulation in wt F2 macrophages and b) following *M. tuberculosis* infection in the lungs *in vivo* (Table S7). Gene ontology and KEGG pathway analysis of these two lists once again identified “immune response” and “antigen processing and presentation” as the key functional annotation (Table S6). This overlap included a strong focus on genes playing a role in antigen presentation by Class I and Class II MHC molecules (*CD74*, *H2-D1*, *H2-DMa*, *H2-DMb1/2*, *H2-Ea*, *H2-Eb1*, *H2-Q8*, *Ltb*, *Tapbp1*), cytokines, chemokines and their receptors (*Ccl6*, *Cxcl9*, *IL6ra*, *Csfr3*, *Fcgrt*, *Tlr9*), anti-viral and anti-bacterial GTPases (*Gbp2,3,5,6*, *Gma1*, *Rgl2*) and other early response genes (*Ifitm1*), as well as a numbers of proteolytic enzymes (*erap1*, lysosyme, endopeptidase) (Figure 4C, Figure 5, and Figure 6).

## Discussion

In this study, we have used transcript profiling and chromatin immunoprecipitation on microarrays (ChIP-chip) to investigate the role of IRF8 in macrophage function, activation by IFNγ/CpG, and response to *M. tuberculosis* infection *in vivo*. For transcript profiling experiments we compared RNA expression profiles from BMDMs obtained from mice that bear either a wt (R294) or a severely hypomorphic *IRF8* allele (C294) derived from the mutant BXH2 mouse strain. In addition, biological and technical RNA replicates were from independent [BALB/c×BXH2] F2 mice of mixed genetic background but genotyped for the two *IRF8* alleles. This was done to increase the stringency of the analysis, and to distinguish true IRF8-dependent effects on gene expression from irrelevant ones resulting from differences in genetic background of the wt (C57BL/6J) and mutant animals (BXH2; mixed C57BL/6J, C3H/HeJ). This approach has been shown to be well suited to map genome-wide eQTLs that segregate as a result of presence or absence of a specific transcription factor [27]–[30]. These experiments produced several lists of genes which levels of expression, under different conditions, is influenced by IRF8. The first list was obtained by comparing BMDMs from wt and *IRF8^C294^* mutant mice, and corresponds to genes which basal level of expression in macrophages is influenced by IRF8 (n = 454). However, because IRF8 plays an important role in maturation of the myeloid lineage [11], [16]–[18], this list may also include genes not directly regulated by IRF8, but rather modulated during macrophages maturation. The second list was obtained by comparing BMDMs from wt and *IRF8^C294^* mutant mice treated with IFNγ/CpG (IRF8 genes regulated during macrophage activation). Two sub-lists were generated, one obtained by pairwise comparison, and the other generated by a 2×2 interaction (Anova) analysis which takes into account IRF8-dependent differences in basal level of expression in absence of IFNγ/CpG stimulation (n = 368). Using ChIP-chip experiments with chromatin prepared from IFNγ/CpG activated macrophages and immunoprecipitated with anti-IRF8 antibodies, we identified a total of 319 IRF8 binding events (minimum of 2 fold enrichment over control ChIP and *p* value≤0.001) on a promoter tiling array. From this information, we further extracted two overlaps and associated critical gene lists. The first one contains 145 genes and corresponds to IRF8 targets (bound by IRF8) that are regulated by exposure to IFNγ/CpG in wt F2 macrophages *in vitro*. The second one contains 359 genes and corresponds to IRF8 targets (bound by IRF8) that are regulated during pulmonary infection with *M. tuberculosis in vivo*. The above-mentioned lists were generated by comparing wt cells to those from BXH2 that bear the severely hypomorphic *Irf8^C294^* allele; nevertheless, small amounts of residual activity may remain in *Irf8^C294^* and gene lists obtained with this mutant may differ somewhat from those obtained by comparing wt cell to cells bearing a null *Irf8^−/−^* allele.

These two gene lists are the most biologically relevant with respect to the role of IRF8 in macrophage function and defenses against *M. tuberculosis in vivo*. A striking feature of these lists is the preponderance of IRF8 targets associated with antigen recognition, processing and presentation by antigen presenting cells (APCs). APCs include dendritic cells, macrophages and B lymphocytes. These cells capture either soluble or particulate antigen by scanning different areas of the body including epithelial surfaces, degrade this antigen and present to T-lymphocytes to activate immune responses. Although virtually all cells can present processed peptide antigens to T cells in association with Class I MHC molecules, so-called “professional APCs” present a wide range of antigens to T cells in association with Class II MHC molecules. Many of the genes coding for proteins involved in antigen recognition, antigen degradation, translocation to a suitable secretory compartment and Class I and Class II molecules are encoded by genes within the major histocompatibility locus (MHC) on mouse Chr. 17 and human Chr. 6.

Presentation of cytosolic peptides via association with Class I MHC molecules occurs in all cells, and is critical for protection against viral infection. It involves proteasome-mediated degradation of viral or other proteins into short peptides, which are then translocated into the endoplasmic reticulum (ER) via the tapasin-associated (Tapap in the ER lumen) ABC transporter heterodimer TAP1/TAP2. Such peptides entering the ER are further trimmed by ER-specific aminopeptidases to fit on the Class I MHC binding site formed by the Class I α chain in association with β2 microglobulin. Peptide-bound Class I complexes are then released from tapasin-chaperone complexes to be delivered to the cell surface, where they can interact with cytolytic CD8^+^ T cells leading to destruction of the infected cells. In addition, Class I MHC antigen presentation is up-regulated by INFα, β, and γ as well as by LT and TNFα. As shown in Figure 5 and Figure 6, we have determined that several genes involved in Class I MHC antigen presentation harbour validated binding sites for IRF8, and/or are regulated in macrophages upon exposure to IFNγ/CpG and/or in the lungs of *M. tuberculosis*-infected mice *in vivo*. These include the PA28 subunit of the proteasome, the TAP1/TAP2 transport system and associated tapasin (Tapap), ER aminopeptidase (Erap1), as well as Class I MHC a chain, β2 microglobulin and associated ER chaperone Calnexin.

On the other hand, antigen presentation via the Class II MHC pathway is carried out by specialized APCs. It involves antigen capture via the endosomal or phagosomal routes through initial interaction with specific cell surface receptors of the TLR (Toll-like receptor), C3R (complement-3 receptor), FcR (Fc receptor) and Ig (immunoglobulin) families. These antigens are digested by members of the cathepsin family of Cys/Asp proteases in acidic endosomes, lysosomes and phagolysosomes. Class II MHC α and β chains stabilized by chaperones are associated with non-polymorphic Ii protein which prevents antigen binding at the α/β interface. Delivery of this complex to antigen-containing acidified endosomes/lysosomes causes proteolytic degradation of Ii (Cd74), leaving only the CLIP (Class II-associated invariant chain peptide) portion of Ii in the antigen binding site. This CLIP peptide is then removed by the HLA-DM (major histocompatibility complex, class II, DM) protein (H2M in mice), freeing up the antigen binding site. Antigen-bound Class II complexes are delivered to the cell surface where they can interact with CD4^+^ helper T cells to induce production of effector T cells, activation of macrophages to microbicidal function, and antibody production depending on the type of APC involved. The process of Class II MHC antigen presentation can itself be stimulated by secretory products of APCs (e.g IL12) or T cells (IFNγ). As shown in Figure 4, Figure 5, and Figure 6, our analysis shows that several genes of the Class II antigen presentation pathway harbour IRF8 binding sites and/or are regulated in macrophages upon exposure to IFNγ/CpG and/or during pulmonary tuberculosis. These include *Tlr4/9/13*, members of the Cathepsin family of proteases, Class II MHC molecules, Ii, and HLA-DM. Together, these results establish a critical role for IRF8 in regulation of the key programmes of antigen presentation in APC. These results are in good agreement with the reported profound defect of IRF8 mutant mice in the DCs compartment, as they lack both CD11c^+^CD8α^+^ DCs and pDCs, and the small number of CD11c^+^CD8α^+^ and CD8α^−^ DCs present in these mice remain immature and fail to up-regulate co-stimulatory molecules and produce key cytokines in response to microbial products *in vitro* and *in vivo* during pulmonary tuberculosis [18], [26]. Macrophage maturation including expression of cytocidal function is also impaired in IRF8-deficient mice, and their macrophages are susceptible to infection with intracellular pathogens *in vitro* [14], [15].

Many of the genes implicated in Class I and Class II MHC antigen presentation are located within the boundaries of the MHC locus. In this locus, we note a striking over-representation of binding sites for IRF8 and the number of genes which expression is regulated by IFNγ/CpG in macrophages. In the case of genes bound by IRF8 and regulated by IFNγ/CpG in macrophages (199 probes, corresponding to 145 genes genome wide), ∼10% of them map to the MHC region on Chr. 17, with 11 binding sites mapping near 16 regulated genes. This concentration of direct IRF8 targets genes corresponds to a 14 fold enrichment over genome-wide representation of these 145 genes. Likewise, ∼10% of the genes regulated by *M. tuberculosis* infection *in vivo* and that contain a IRF8 binding site in their vicinity map to the MHC locus (9 fold enrichment), with 12 IRF8 binding sites mapping near 27 regulated genes (Figure 5). The regulatory role of IRF8 in the MHC locus also seems to include additional genes playing a central role in amplification of early immune response, such as *TNFα*, *LT*, and components of the complement pathway. IRF8-dependent transcription of MHC-linked genes may involve direct cis-acting effects of IRF8 binding to *de novo* motifs identified in our study, or may additionally involve amplification through activation of other transcription factors. For example, Ciita (class II, major histocompatibility complex, transactivator) is a non-DNA binding co-activator that binds to the so-called “MHCII enhanceosome” multiprotein complex and that serves as a master control factor for MHCII gene expression [44]. *Ciita* expression is up-regulated by IFNγ in a STAT1-dependent fashion; through the presence of GAS (Gamma interferon activation site) element in the proximal PIV responsive promoter in the *Ciita* gene [45]. Moreover, we observed that *Ciita* expression is tightly regulated by IFNγ/CpG in an IRF8-dependent fashion in macrophages from F2 mice (2×2 interaction Anova analysis; Table S3), and is also regulated in pulmonary tuberculosis. The PIV promoter region of *Ciita* also contains a *de novo* IRF8 binding motif, and a cluster of weak binding sites were experimentally detected in this region by ChIP-chip (data not shown). Therefore, it is possible that IRF8-mediated control of MHC gene expression involves amplification by other transcriptional regulators such as Ciita.

Furthermore, our study points at an important role of IRF8 in transcriptional activation of several families of IFN-inducible intracellular GTPases of the p47 (IRG), p65 (GBPs) and Dynamin (Mx) families which are known to be essential for protection against intracellular bacterial, parasitic and viral infections (Figure 4C) [40]. The p47 family of immunity-related GTPase (IRGs) contains 18–23 members in mice, with 6 having been characterized in some details [40], [41]. They are expressed at low levels in different cell types, but mainly myeloid cells, and show dramatic up-regulation upon exposure to IFNγ. Studies in mutant mice have shown that deficiency in Lrg47/Irgm1 causes susceptibility to infection with *M. tuberculosis* [46], [47], while absence of Igtp/Irgm3 and Iigp1/Irga6 causes intracellular replication of *Toxoplasma gondii* [48], [49]. In macrophages, the Irgm1 protein is rapidly recruited to the membrane of bacteria-containing phagosomes, where it is believed to facilitate delivery of lysosomal cargo for the destruction of intracellular pathogens, a process that is critically dependent on phosphatidylinositol 3,4 bisphosphate (PI3,4P_2_) and PI3,4,5P_3_ [50]. As expected, we observed increased expression of several IRGs in macrophages in response to IFNγ and *in vivo* in *M. tuberculosis* infected lungs (Figure 4C), but we also detected at least one IRF8 binding site near Irgm1, with two weaker sites near Irg-47 and Irgm3. Unfortunately, several of the IRGs promoter regions were not present on the arrays we used, and more experimentation will be required to determine if IRF8 binding sites are present at or near other IRG genes. The family of p65 GBP contains 11 members in mice that map to two gene clusters on chromosomes 3 (Gbp1, 2, 3, 5, 6, 13) and 5 (Gbp4, 8, 9, 10, 11, 12) [39], [41]. Gbp mRNAs are induced by IFNγ in macrophages *in vitro*, and in spleen, liver and lungs of mice infected with intracellular pathogens *Listeria monocytogenes* and *T. gondii* [39]. Subcellular localization studies have shown that several Gbp family members are quickly recruited to the membrane of microbe-containing phagosomes formed in infected fibroblasts [39]. Like Irg proteins, Gbps also traffic to pathogen vacuoles to potentially deliver microbicidal products to restrict intracellular replication. We have detected by ChIP-chip 3 IRF8 binding sites on the chromosome 3 cluster that are associated with regulation of several of these Gbps (Gbp2, 3, 5, 6) in response to IFNγ or *M. tuberculosis* infection. We have validated at the protein level the IRF8-dependence of basal and IFNγ/CpG inducible expression of Cd74 (and its cleavage product SLIP) (Figure 6B), and Gbp1 in macrophages (Figure S2), with more modest effects noted at the protein level for Gbp2, Gbp3 and Irgm 1 (Figure S2). Although transcriptional activation of the Irg and Gbp genes by IFNγ was previously associated with the presence of GAS (Gamma interferon activation site) and ISRE elements in their promoter region, and activation via the Jak/Stat pathway and IRF1 [41], our results strongly suggest that IRF8 may additionally be involved in this regulation. Moreover, the study of *Irgm3^−/−^* mutant mice has identified defects in antigen cross-presentation in these mice [51]. Interestingly, it has been proposed that IRG proteins (and possibly Gbps) may not only be involved in the delivery of lysosomal cargo to bacterial-containing phagosomes, but may also be involved in facilitating transport of antigen containing lipid droplets for antigen cross-presentation by Class I MHC molecules [51]. Although speculative, this proposal is in agreement with the observed role of IRF8 in directing transcriptional networks associated with antigen presentation by Class I and Class II MHC molecules.

Finally, a recent study [31] used a combination of IRF8 ChIP-chip and expression profiling in IRF8 knocked down human myelomonocytic leukemia THP-1 cells to identify primary and secondary IRF8 targets in these cells. In agreement with the *de novo* IRF8 binding motif described herein (Figure 3A and Figure S1A), previous gene specific studies [52]–[55] and our binding motif association study (Figure 3C), these authors demonstrated a significant overlap between IRF8 and PU.1 ChIP-chip binding locations. They identified development and differentiation genes affected by the loss of IRF8, but also immune response genes as direct targets. The list of 84 IRF8 primary targets was compared to our results (Table S8). This list shows a 43% overlap with our list of IRF8 binding peaks, a 21% overlap with our list of genes regulated by IFNγ/CpG in a IRF8-dependent fashion, and 70% overlap with the list of genes differentially regulated during pulmonary tuberculosis *in vivo*. Therefore, the overlaps between the IRF8 target genes identified in both studies is fairly important. Together, these studies emphasize the predominant role of IRF8 in myeloid cell functions.

## Materials and Methods

### Animals

C57BL/6J (B6) mice were purchased from the Jackson Laboratory (Bar Harbor, ME). Recombinant inbred BXH2 mice were originally derived by B. Taylor at the Jackson Laboratory [56] and subsequently maintained as a breeding colony at McGill University. BXH2 males were used to generate [BALB/cJ×BXH2]F_1_ mice, which were then inter-crossed to produce an F2 progeny. F2 littermates homozygote for either the wt (R294; *IRF8^+/+^*) or mutant (C294; *IRF8^R294C/R294C^*) *IRF8* allele were identified and used in the transcript profiling experiments. *IRF8* alleles were identified by genotyping for the proximal marker *D8Mit13* using oligonucleotide primer pairs 5′-CCTCTCTCCAGCCCTGTAAG-3′ and 5′-AACGTTTGTGCTAAGTGGCC-3′, which distinguishes between BALB/cJ and C57BL/6J, the strain background of the *IRF8* genomic segment onto which the R294C mutation appeared in BXH2 [9]. The isolation of genomic DNA, and the genotyping for *D8Mit13* alleles were carried out as described [9]. Male and female mice 8 to 12 weeks of age were used for all experiments, according to guidelines and regulations of the Canadian Council on Animal Care.

### Macrophages and Stimulation

The mouse macrophage cell line J774 was grown in Dulbecco's modified Eagle's medium (DMEM, Sigma) supplemented with 10% heat-inactivated fetal bovine serum (HI-FBS, GIBCO), 100 U/ml penicillin, and 50 µg/ml streptomycin (Invitrogen) at 37°C, in 5% CO_2_-containing humidified air. BMDMs were isolated from femurs of 8- to 12-week-old mice and were cultured in DMEM (Sigma) containing 10% heat-inactivated fetal bovine serum (HI-FBS), 20% L-cell-conditioned medium (LCCM), 100 U/ml penicillin, and 100 µg/ml streptomycin in bacteriological grade dishes (Fisher) at 37°C in a humidified atmosphere containing 5% CO_2_. Seven days later, cells were harvested by gentle washing of the monolayer with phosphate-buffered saline containing citrate. Cells were plated in 150-mm tissue culture-grade plastic plates (18×10^6^ cells per plate; Corning) in DMEM containing 10% HI-FBS, 10% LCCM, 100 U/ml penicillin, and 100 µg/ml streptomycin. In some experiments, macrophages were primed with IFNγ (50 U/ml) for 18 hrs, prior to stimulation (3 hrs) with recombinant mouse IFNγ (Cell Sciences, Canton, MA), and CpG DNA oligonucleotides (5′-TCCATGACGTTCCTGACGTT-3′) used at a concentration of 400 U/ml and 1,5 µg/ml, respectively. IFNγ and CpG stimulate both the IFNγ receptor and Tlr9, and engagement of both receptors stimulates IRF8 expression, via STAT1, NFKB and possibly other pathways [11].

### Transcript Profiling with Microarrays

Total RNA was extracted from BMDMs obtained from 6 individual mice per experimental group, either prior to or 3 hrs following stimulation of BMDMs with IFNγ (400 U/ml) and CpG DNA (1,5 µg/ml). IFNγ/CpG-stimulated macrophages were initially primed with IFNγ (50 U/ml) for 18 hrs. Purified RNAs were analyzed for integrity by gel electrophoresis, and were then hybridized to microarrays (Illumina Mouse WG-6 v2.0 Expression BeadChip) according to the manufacturer's recommended experimental protocol. To minimize technical variability, RNA processing steps (RNA extraction, probe labeling and microarray hybridization) were executed in parallel for all samples. The GeneSifter™ microarray data analysis system (Geospiza Inc., Seattle, WA, USA) was used to examine data generated from comparisons between control (unstimulated) and IFNγ/CpG-stimulated (3 hrs) groups. Log transformed data were normalized and transcripts showing differential expression were identified by pairwise, or two-way (2×2 interaction) Anova analysis with a *t* test p value of 0.05 and a fold-change cutoff of 1.5X. Hierarchical clustering based on complete linkage method was applied to evaluate the effect of the different sources of variability (IRF8 alleles, treatments, host specific responses). Complete microarray data (accession no. E-MEXP-2962) has been deposited in the ArrayExpress database (www.ebi.ac.uk/microarray-as/ae/).

### Quantitative PCR

The expression of individual mRNAs was measured by quantitative PCR (qPCR) amplification of cDNA transcripts generated by reverse transcriptase (RT). Briefly, RNA samples (n = 6) used for transcriptional profiling were pooled and 3 µg of pooled RNA was converted to cDNA using Moloney murine leukemia virus (Invitrogen) in a 20 µl reaction according to the manufacturer's recommended experimental protocol. PCR amplification was performed using Quantitech SYBR Green PCR kit (Qiagen), and all samples were measured in duplicate. Each reaction contained 2 µl of cDNA template, 1 µl of the target-specific primer pair (each primer at 5 µM), 9.5 µl of RNase-free water and 12.5 µl of Quantitech SYBR Green PCR master mix. PCR amplification included an initial denaturation step (10 min at 95°C) followed by 50 cycles of amplification (15 s at 95°C, 30 s at 57°C, and 33 s at 72°C), and was performed using the 7500 Real Time PCR system (Applied Biosystems). PCR primers were designed to generate amplicons ranging from 100 to 150 bp. The *Hprt* gene was used as a constitutively expressed internal control to normalize the mRNA levels of target genes.

### Western Blot Analysis

BMDMs were obtained from wt (B6) and IRF8 mutant (BXH2) mice, either prior to or following stimulation (4 and 24 hrs) with IFNγ (400 U/ml) and CpG DNA (1,5 µg/ml). Whole cell extracts (75 µg per lane) were subjected to 10% sodium dodecyl sulfate–polyacrylamide gel electrophoresis (SDS–PAGE), followed by electroblotting and overnight incubation with the monoclonal anti-Cd74 (Ii) antibody (clone In-1 purchased from BD Pharmingen) (used at 1∶200). Immune complexes were revealed with a horseradish peroxidase-conjugated goat anti-rat antibody (used at 1∶3000) and visualized by enhanced chemiluminescence (SuperSignal West Pico kit, Thermo Scientific, Rockford, IL). Intact p41 and p31 Ii, as well as SLIP Ii fragment (contains the NH_2_-terminal portion of Ii) are all detected by the In-1 mAb [57]. Antibodies, dilutions and source dilutions for the immune GTPases were: Irgm1 (A19, 1∶200), Irgm3 (M14, 1∶200), Irga6 (G20, 1∶200), Irgb6 (A20, 1∶200), Gbp1 (M18, 1∶200), Gbp2 (M15, 1∶1000), Gbp5 (L12, 1∶500) were from Santa Cruz Biotechnology; Gbp3 (Abcam, 1∶200), Beta actin (Sigma, 1∶1000).

### Chromatin Immunoprecipitation (ChIP)

ChIP assays were performed on J774 macrophages stimulated with IFNγ/CpG for 3 hours, according to a method previously described [58], [59]. Stimulated cells were treated with formaldehyde (1% final; 10 min, 20°C), washed with ice-cold PBS, and cross-linked cells were harvested by centrifugation. The cell pellet was resuspended in 1 mL of cell lysis buffer (1% SDS, 10 mM EDTA, 50 mM Tris-HCl pH 8) supplemented with a cocktail of protease inhibitors, followed by sonication on ice. Chromatin was recovered by centrifugation (13,000 g, 7 min, 4°C), and resuspended in ChIP dilution buffer (0.5% Triton X-100, 2 mM EDTA, 100 mM NaCl, 20 mM Tris-HCl pH 8.1) followed by pre-clearing using a 50% slurry of salmon sperm DNA/protein G agarose beads (Upstate/Millipore) for 2.5 hrs at 4°C. IRF8-DNA complexes were immunoprecipitated (4°C, 16 hrs) using an anti-IRF8 antibody (sc-6058x; Santa Cruz), followed by addition of 50% slurry of salmon sperm DNA/protein G beads (600 µL; 3 hr, 4°C) on a rotating device. Control and anti-IRF8 immunoprecipitates were washed (10 min) sequentially with each of the following buffers: low salt Buffer I (1% Triton X-100, 0.1% SDS, 150 mM NaCl, 2 mM EDTA pH 8.0, 20 mM Tris-HCl pH 8.1), high salt Buffer II ( 1% Triton X-100, 0.1% SDS, 500 mM NaCl, 2 mM EDTA pH 8.0, 20 mM Tris-HCl pH 8.1) and Buffer III (1% IGEPAL, 0.25 mM LiCl, 1% Na-deoxycholate, 1 mM EDTA pH 8.0, 10 mM Tris-HCl pH 8.1), and a brief final wash in TE buffer (10 mM Tris-HCl pH 7.5, 1 mM EDTA pH 8). DNA was recovered from immunoprecipitated IRF8 chromatin complexes by incubation in a buffer containing 1% SDS and 0.1 M NaHCO_3_ (65°C, 16 hrs), and further purified using the QIAquick PCR purification kit (Qiagen).

### Sample Preparation for Hybridization to Mouse Extended Promoter Arrays (ChIP-Chip)

Sample preparation for hybridization to promoter arrays was carried out as recommended in Agilent Mammalian ChIP-on-chip protocol, with minor modifications. Briefly, the ChIP DNA was amplified by ligation-mediated PCR (LM-PCR) following DNA blunting and linker ligation. The LM-PCR samples were purified on QIAquick purification columns and submitted to 18 additional rounds of amplification in the presence of aminoallyl-dUTP (final concentration 300 µM; Sigma). The LM-PCR samples containing aminoallyl-dUTP were purified (QIAquick PCR purification columns) and labeled with Cy3 and Cy5 dies. The DNA amount was calculated by using the OD at 260 and 320, and the Cy3 and Cy5 incorporation was also determined.

### Agilent ChIP-Chip Hybridization and Analysis

Samples were hybridized to Agilent 244K mouse extended promoter arrays containing ∼17,000 of the best-defined mouse transcripts as defined by RefSeq spanning the regions from −5.5 kb upstream to +2.5 kb downstream of the transcription start site. The procedure was done according to the Agilent mammalian ChIP on chip protocol version 9.2. Following the hybridization at 65°C for 40 hrs, the arrays were washed and scanned using a GenePix 4000B scanner and data was extracted from the images using Agilent Feature Extraction software as described in the mammalian ChIP on chip protocol (Agilent, v.10). Data from ChIP-chips were normalized and averaged using ChIP Analytics 1.3 software. Data was processed in ChIP Analytics using the intra-array Lowess normalization, Whitehead Error Model v1.0 and Whitehead Per-Array Neighbourhood Model v1.0 for peak detection and evaluation. The default parameters were used to identify significant binding events (1000 bp as the maximum distance for 2 probes to be considered neighbors in a probe set, probe set p-value<0.001 for a “bound” probe).

### Transcription Factor Binding Motifs Analyses

We retrieved from the UCSC genome browser 500 bp sequences centered on each 319 IRF8 ChIP-chip and performed *de novo* binding motif analyses with 3 different algorithms: MEME [32], MDscan [33] and AlignACE [34]. The resulting matrices were compared to the Transfac v11.3 known binding motif database using the STAMP web-tool [35]. The schematic representations of the IRF8 *de novo* binding motif were generated with WebLogo [60]. The 319 IRF8 binding regions were queried for all known binding motifs on 1000 bp sequences using the optimized matrix threshold from MatInspector software (Genomatix). Then, we searched for the same motifs on five sets of 319 randomly chosen 1000 bp sequences, selected from Agilent 244K mouse extended promoter array oligos. Thereafter, we calculated enrichment of binding motifs between the IRF8 binding regions and the mean of motif occurrence in random sequence sets (Table S5).

### Gene Ontology (GO) and Pathway Analyses

We used the DAVID (database for annotation, visualization and integrated discovery) website to calculate GO and KEGG (Kyoto encyclopedia of genes and genomes) pathways enrichment in our different ChIP-chip and expression datasets (DAVID threshold set to p value≤0.001) [36], [37]. The Agilent 244K mouse extended promoter array or Illumina Mouse WG-6 v2.0 Expression BeadChip complete gene lists were used as reference respectively for enrichment evaluation.

## Supporting Information

Figure S1 De novo transcription factor binding motif analyses on IRF8 ChIP-chip binding sites. (A) 500 bp of sequence flanking the 319 IRF8 binding peaks were queried for *de novo* motif finding with three different algorithms: MEME, MDscan and AlignACE [32]–[34]. The top motifs returned by each algorithm are highly similar. (B) Weight matrix representation of the known Transfac IRF8 (Icsbp) binding motif.
(TIF)

Figure S2 Validation of members of the Gbp (p65) and Irgm (p47) families at protein level. BMDMs total proteins were obtained from wt (B6) and IRF8 mutant (BXH2) mice either prior to (unstimulated control) or following stimulation with IFNγ/CpG (4 hrs and 24 hrs post-stimulation), separated in 10% SDS-PAGE (35 µg of lysate/lane) and probed with specific antibodies. β-actin was used as a constitutively expressed internal control to normalize the protein levels.
(TIF)

Table S1 Genes differentially regulated at basal level in wt versus IRF8 mutant macrophages (pairwise analysis).
(XLS)

Table S2 Genes differentially regulated in wt versus IRF8 mutant macrophages at basal level, and modulated by IFNγ/CpG stimulation (pairwise analysis).
(XLS)

Table S3 Genes differentially modulated by IRF8 in wt versus IRF8 mutant BMDMs in response to IFNγ/CpG exposure (two-way Anova; 2×2 interaction).
(XLS)

Table S4 List of IRF8 ChIP-chip binding sites.
(XLS)

Table S5 Known transcription factor binding motifs enrichment analysis.
(XLS)

Table S6 Gene Ontology and KEGG pathways enrichment analysis.
(XLS)

Table S7 Intersection between IRF8 ChIP-chip binding sites and differentially regulated genes in IFNγ/CpG treated F2 mice or *M. tuberculosis*-infected B6 mice for 30 days.
(XLS)

Table S8 Intersection between IRF8 target lists from Ref. [31] and those from the present study.
(XLS)
