# Supplementary figures and images for: Interferon Regulatory Factor 8 Regulates Pathways for Antigen Presentation in Myeloid Cells and during Tuberculosis
Source: PLoS Genet. 2011 Jun 23;7(6):e1002097. doi: 10.1371/journal.pgen.1002097 (PMC3121741; doi:10.1371/journal.pgen.1002097)

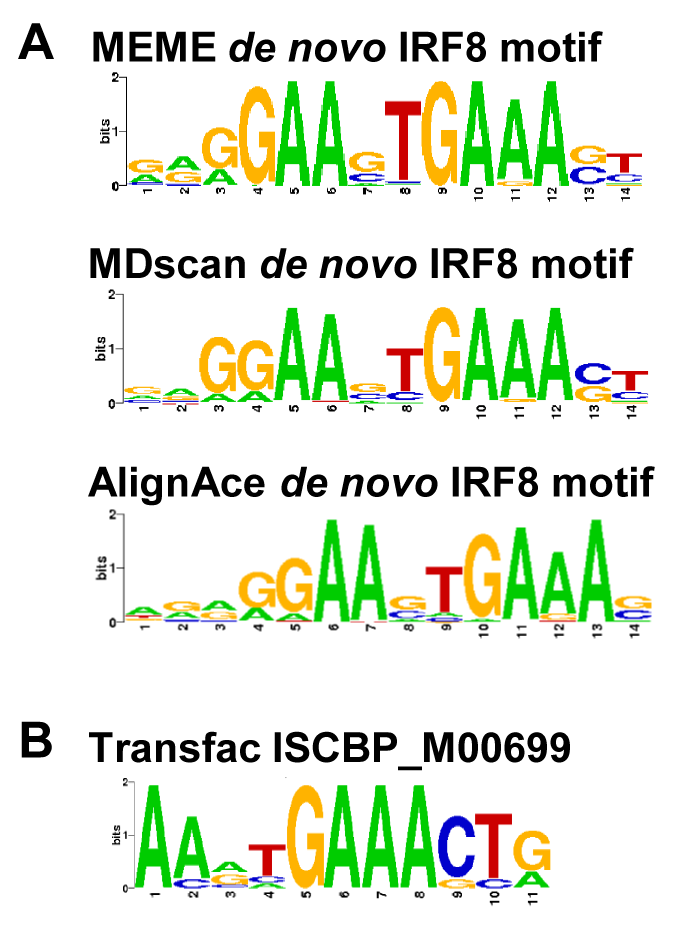

Supplement: Figure S1 — De novo transcription factor binding motif analyses on IRF8 ChIP-chip binding sites. (A) 500 bp of sequence flanking the 319 IRF8 binding peaks were queried for de novo motif finding with three different algorithms: MEME, MDscan and AlignACE [32]–[34]. The top motifs returned by each algorithm are highly similar. (B) Weight matrix representation of the known Transfac IRF8 (Icsbp) binding motif. (TIF) [file pgen.1002097.s001.tif]

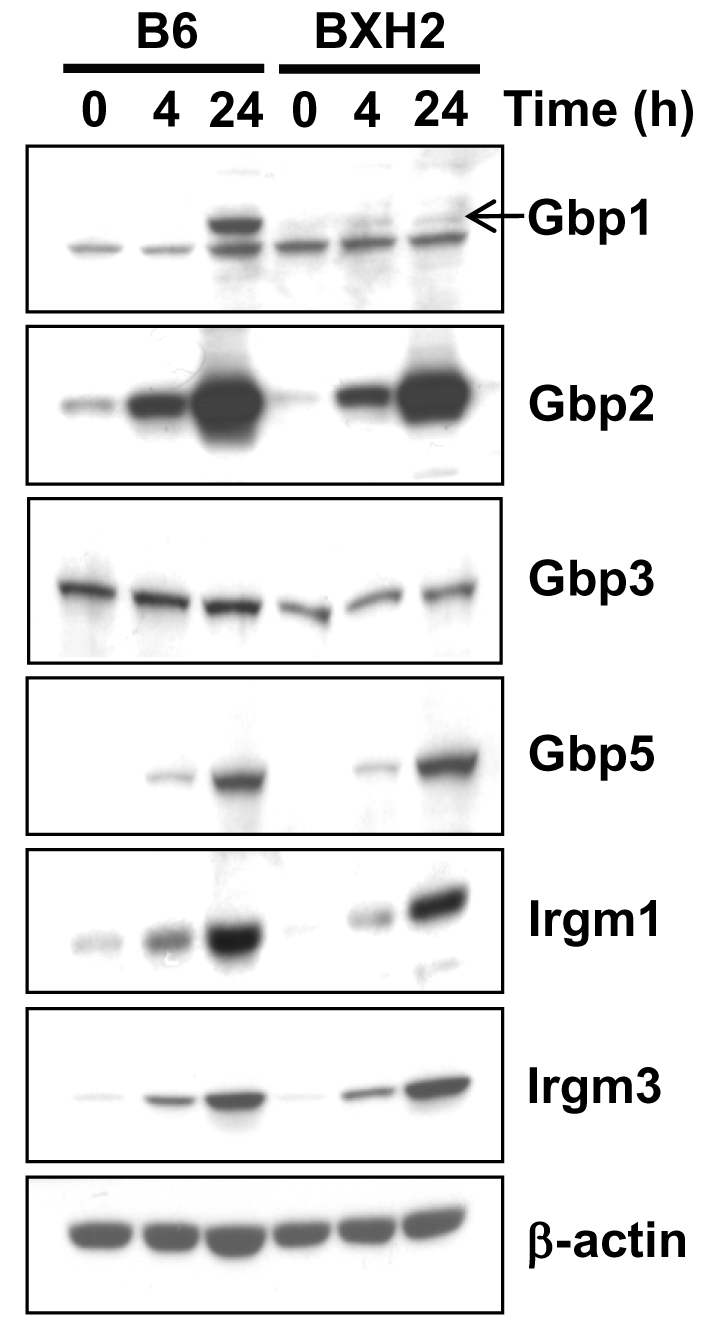

Supplement: Figure S2 — Validation of members of the Gbp (p65) and Irgm (p47) families at protein level. BMDMs total proteins were obtained from wt (B6) and IRF8 mutant (BXH2) mice either prior to (unstimulated control) or following stimulation with IFNγ/CpG (4 hrs and 24 hrs post-stimulation), separated in 10% SDS-PAGE (35 µg of lysate/lane) and probed with specific antibodies. β-actin was used as a constitutively expressed internal control to normalize the protein levels. (TIF) [file pgen.1002097.s002.tif]
